# Endosymbiont population genomics sheds light on transmission mode, partner specificity, and stability of the scaly-foot snail holobiont

**DOI:** 10.1038/s41396-022-01261-4

**Published:** 2022-06-17

**Authors:** Yi Lan, Jin Sun, Chong Chen, Hao Wang, Yao Xiao, Maeva Perez, Yi Yang, Yick Hang Kwan, Yanan Sun, Yadong Zhou, Xiqiu Han, Junichi Miyazaki, Tomo-o Watsuji, Dass Bissessur, Jian-Wen Qiu, Ken Takai, Pei-Yuan Qian

**Affiliations:** 1 Southern Marine Science and Engineering Guangdong Laboratory (Guangzhou), Guangzhou, China; 2 Department of Ocean Science, The Hong Kong University of Science and Technology, Hong Kong, China; 3 Institute of Evolution & Marine Biodiversity, Ocean University of China, Qingdao, 266003 China; 4 X-STAR, Japan Agency for Marine-Earth Science and Technology (JAMSTEC), 2-15 Natsushima-cho, Yokosuka, 237-0061 Kanagawa Prefecture Japan; 5 Department of Biological Sciences, University of Montreal, Montreal, Quebec, Canada; 6 Department of Biology and Hong Kong Branch of the Southern Marine Science and Engineering Guangdong Laboratory (Guangzhou), Hong Kong Baptist University, Kowloon Tong, Hong Kong; 7 Key Laboratory of Marine Ecosystem Dynamics & Second Institute of Oceanography, Ministry of Natural Resources, Hangzhou, 310012 China; 8 Key Laboratory of Submarine Geosciences & Second Institute of Oceanography, Ministry of Natural Resources, Hangzhou, 310012 China; 9 Department of Food and Nutrition, Higashi-Chikushi Junior College, 5-1-1 Shimoitozu, Kitakyusyu, 803-0846 Japan; 10 Department for Continental Shelf, Maritime Zones Administration & Exploration, Prime Minister’s Office, 2nd Floor, Belmont House, 12 Intendance Street, Port Louis, 11328 Mauritius

**Keywords:** Bacterial genomics, Marine microbiology, Symbiosis

## Abstract

The scaly-foot snail (*Chrysomallon squamiferum*) inhabiting deep-sea hydrothermal vents in the Indian Ocean relies on its sulphur-oxidising gammaproteobacterial endosymbionts for nutrition and energy. In this study, we investigate the specificity, transmission mode, and stability of multiple scaly-foot snail populations dwelling in five vent fields with considerably disparate geological, physical and chemical environmental conditions. Results of population genomics analyses reveal an incongruent phylogeny between the endosymbiont and mitochondrial genomes of the scaly-foot snails in the five vent fields sampled, indicating that the hosts obtain endosymbionts via horizontal transmission in each generation. However, the genetic homogeneity of many symbiont populations implies that vertical transmission cannot be ruled out either. Fluorescence in situ hybridisation of ovarian tissue yields symbiont signals around the oocytes, suggesting that vertical transmission co-occurs with horizontal transmission. Results of in situ environmental measurements and gene expression analyses from in situ fixed samples show that the snail host buffers the differences in environmental conditions to provide the endosymbionts with a stable intracellular micro-environment, where the symbionts serve key metabolic functions and benefit from the host’s cushion. The mixed transmission mode, symbiont specificity at the species level, and stable intracellular environment provided by the host support the evolutionary, ecological, and physiological success of scaly-foot snail holobionts in different vents with unique environmental parameters.

## Introduction

Eukaryotes establishing a symbiotic relationship with chemosynthetic bacteria are widespread among diverse marine habitats, from shallow water to deep sea [1]. For example, solemyids, lucinids, and thyasirids which dwell in both shallow-water and deep-sea chemosynthetic environments harbour chemosynthetic symbionts [1]. Deep-sea chemosynthetic environments, such as hydrothermal vents and hydrocarbon seeps, are the best known habitats for chemosymbiosis. In these ecosystems, many endemic animals depend on the chemosynthesis of symbiotic microbes for nutrients. For instance, *Bathymodiolus* mussels, *Alviniconcha* snails, and *Riftia* tubeworms acquire environmental bacteria via horizontal transmission upon settlement and retain them as endosymbionts [2–4]. Deep-sea vesicomyid clams, such as *Phreagena okutanii* and *Turneroconcha magnifica*, rely on vertical transmission to transmit their endosymbionts from mother to offspring [5–7]. Solemyid clams, such as *Solemya velum*, adopt a combination of vertical and horizontal transmission to obtain their symbionts [8, 9]. However, the life cycles and transmission modes of most other deep-sea chemosymbiotic organisms are unknown.

Transmission mode plays a pivotal role in the symbiosis establishment, partner specificity, and symbiont genetic diversity of a holobiont. Stringent vertical transmission leads to strong genetic coupling between the host mitochondria and symbionts because they are transmitted together to the offspring via gametes over generations [7]. Conversely, in horizontal transmission, the coupling of alleles between the host mitochondria and symbionts can be obscured [8, 9]. For example, the phylogeny of the host mitochondria and symbionts of many vesicomyid clams adopting vertical transmission is congruent [10]. However, occasional horizontal transmission events, such as those in *T. magnifica* and *Calyptogena fausta* clams, have been shown to break the phylogenetic congruency between host mitochondria and symbionts [9, 11]. *Bathymodiolus* mussels using horizontal transmission and *Solemya* clams using a mixed transmission mode, on the other hand, exhibit incongruent phylogeny with their symbionts [9].

Another theoretical assumption is that the environmental pool of symbionts is genetically diverse but homogeneous within a small-scale area, where different host individuals have access to the same symbiont strains [3]. Under this assumption, if horizontal transmission persists for at least a significant part of the lifetime of individual hosts, the genetic diversity of symbiont populations could closely reflect that of the environmental pool and exhibit high intra-host heterogeneity and inter-host homogeneity across host individuals living in the same vent field [2]. For instance, distinct strains of specific bacterial species can co-exist in *Bathymodiolus* mussels, and the symbiont strain heterogeneity is similar in mussels living in the same vent field but different across different vents [2]. However, if horizontal transmission occurs in a restricted process (e.g. a short period of time in the life cycle of the host or limited uptake of symbionts), the symbiont populations would be affected by strong population bottlenecks [6, 12]. Limited strains  taken up by the host would lead to intra-host homogeneity, which has been observed in *Riftia*, *Escarpia*, and *Lamellibrachia* tubeworms [13, 14]. If the host individuals transfer symbionts in a short period and the free-living symbionts change under fluctuating vent environments, the inter-host heterogeneity would increase across host individuals. This phenomenon has been observed in *B. brooksi* mussels [12]. In vertical transmission, only a small part of symbionts would be transmitted with egg cells, and genetic drift further lowers the intra-host symbiont genetic diversity. The accumulation of within-host mutations increases the divergence of symbiont populations across host individuals [6]. In this case, symbiont populations are expected to present extreme intra-host homogeneity and inter-host heterogeneity. Therefore, different transmission modes dominating in each lineage would generate genetic signatures over evolutionary time scale, and population genomic tools can be used to study the genetic associations between host mitochondria and symbionts as well as intra-host and inter-host symbiont populations to understand their transmission mode [10].

The scaly-foot snail *Chrysomallon squamiferum* is a peltospirid vent snail distributed in the Indian Ocean and houses a sulphur-oxidising gammaproteobacteria as the endosymbiont [15]. Vent molluscs normally house symbionts in the gill that is in direct contact with vent fluid, but the scaly-foot snail hosts the endosymbiont in a hypertrophied oesophageal gland deep inside its body and has a much enlarged circulatory system which delivers sulphide and carbon dioxide to the endosymbiont [16]. Previous analyses of its hologenome showed that the symbiont can utilise sulphuric substances from the vent fluid and synthesise nutrients for the host [15, 17]. The endosymbiont genome also possesses a hydrogenase gene cluster for hydrogen oxidation [15], but whether the endosymbiont of scaly-foot snails can utilise hydrogen remains unclear, similar to the symbiont of *Riftia* tubeworms which were unable to utilise hydrogen under experimental conditions, even though they possess hydrogenase genes [18].

A previous study of scaly-foot snails reported high genetic homogeneity in the endosymbiont population but divergent mitochondrial genes amongst host individuals from the Kairei vent field on the Central Indian Ridge (CIR), suggesting that this species adopts horizontal transmission [15]. Previous population genetic studies on scaly-foot snail populations from Kairei and Solitaire vent fields on the CIR as well as Tiancheng and Longqi vent fields on the Southwest Indian Ridge (SWIR) revealed that these populations are well connected, with the exception of Longqi, and the snails exhibit considerable genetic diversity within each vent field [19, 20]. Whether the endosymbiont genetic homogeneity across the host individuals in Kairei [15] also persists in populations from other vent fields where the endosymbiont has not been studied at the population level remains unclear. This topic is of interest because the transmission mode, symbiont genetic diversity, and adaptation of endosymbionts are poorly studied, especially in Indian Ocean vents.

In the present study, we use population genomics to compare the endosymbionts of scaly-foot snails from five vent sites: Kairei and Solitaire fields on CIR, Tiancheng and Longqi fields on the SWIR, and a newly discovered population in Wocan field on the Carlsberg Ridge (CR) in the northern Indian Ocean [21]. Specifically, we explore the transmission mode, partner specificity, and stability of host–symbiont associations in scaly-foot snails across the five vent fields separated by a maximum linear distance of approximately 5000 km (i.e. between Longqi and Wocan). Considering the results of the present study where the genetic divergence between symbiont populations from the Kairei and Solitaire vents are relatively low and therefore useful to test how the symbiosis functions under different environments, we further examine how the scaly-foot snail holobiont copes with different environmental conditions in the two vents. In situ environmental factors and gene expression patterns of in situ fixed individuals from colonies at Kairei and Solitaire fields are measured to determine their variations between vents and fluctuations within a colony.

## Materials and methods

### Deep-sea sampling

**Fig. 1:**
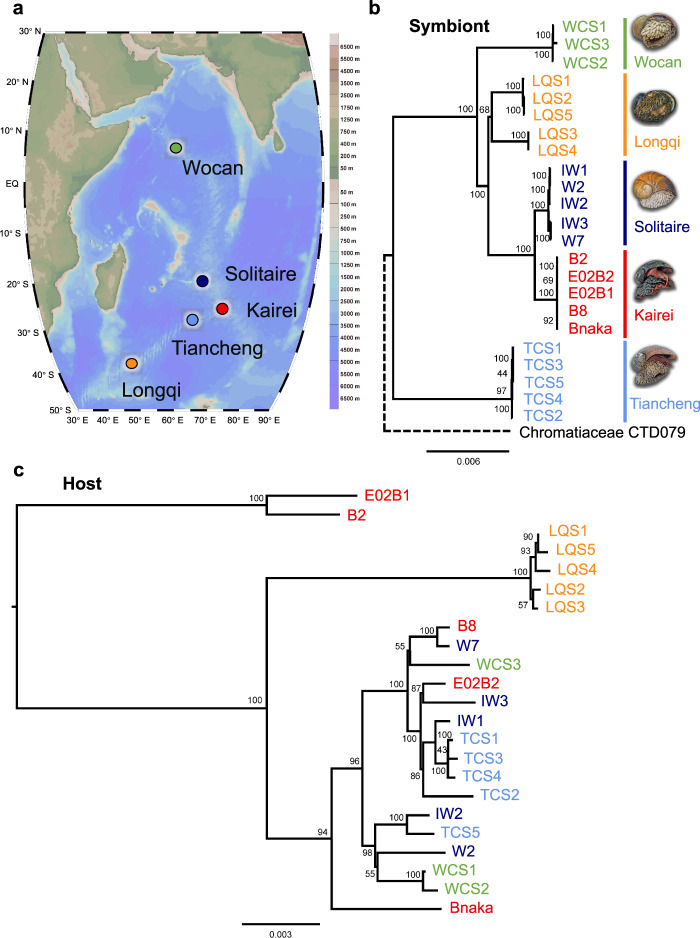
Phylogeny of the scaly-foot snail holobiont from five hydrothermal vents in the Indian Ocean. **a** Bathymetric map showing the relevant Indian Ocean hydrothermal vent fields on the Carlsberg Ridge (Wocan marked in green dot), the Central Indian Ridge (Solitaire in dark blue dot and Kairei in red dot), and the Southwest Indian Ridge (Tiancheng in light blue dot and Longqi in orange dot). **b** Phylogenetic tree based on 1499 single-copy core genes of scaly-foot snail endosymbionts from the five vent fields. The Chromatiaceae bacterium CTD079 served as an outgroup (Sass et al. 2020 [35]). **c** Phylogenetic tree of host scaly-foot snails based on 11 protein-coding genes of the mitochondrial genome from the five vent fields. Wocan (green): WCS1, WCS2, and WCS3; Solitaire (dark blue): IW1, IW2, IW3, W2, and W7; Kairei (red): B2, B8, E02B1, E02B2, and Bnaka (from Nakagawa et al. 2014 [15]); Tiancheng (light blue): TCS1, TCS2, TCS3, TCS4, and TCS5; Longqi (orange): LQS1, LQS2, LQS3, LQS4, and LQS5. Source data of (**a**) is provided in a Source Data file.

Scaly-foot snails were collected from Longqi (“Tiamat” chimney; 37.7839°S, 49.6502°E; 2761 m depth; cruise COMRA DY52; April 2019) and Tiancheng (“Tiantang” chimney; 27.8508°S, 63.9227°E; 2705 m; cruise COMRA DY52; April 2019) on the SWIR by the remotely operated vehicle (ROV) *Sea Dragon III* on-board R/V *Dayang Yihao*, from Solitaire (19.5568°S, 65.8482°E; 2606 m depth; cruise YK13-02; February 2013) and Kairei (25.32°S, 70.0403°E; 2415 m depth; cruise YK13-03; March 2013; and YK16-E02; February 2016) on the CIR by the manned submersible DSV *Shinkai* 6500 on-board R/V *Yokosuka* and from Wocan (60.53°E, 6.36°N; 2919 m; cruise COMRA DY38; March 2017) on the CR by the HOV *Jiaolong* on-board R/V *Xiangyanghong 9* (Fig. 1a). Snails collected from all sites were immediately stored at −80 °C upon recovery on-board until further usage. A few additional snails from Kairei and Solitaire were fixed in situ using RNA Stabilization Reagent. In detail, during sampling in the seafloor, the snails were collected using a suction pump sampler and immersed in a RNA Stabilization Reagent (25 mM sodium citrate, pH 5.2, 10 mM EDTA, 0.7 kg/L ammonium sulphate) that filled the suction chamber [22, 23]. The sampling system included a collapsible polyethylene bag (Rontainer, Sekisui Chemical, Osaka, Japan) with 10 L of RNA Stabilization Reagent positioned above the suction chamber (5 L in volume) to which it was connected via a tube through a ball valve. After the snails were collected into the suction chamber, the valve was opened and a 5 kg lead weight was placed on top of the collapsible plastic bag to force the RNA Stabilization Reagent into the sampling chamber by gravity. As the RNA Stabilization Reagent is much denser than seawater, it eventually substituted the seawater completely and fixed the snails. The snails remained in the RNA Stabilization Reagent until recovery on-board. Upon recovery on-board, the snails were dissected. The tissues were stored in fresh RNAlater until sufficient infiltration and finally frozen at −80 °C. A few individuals from the Solitaire vent were fixed in 4% paraformaldehyde (PFA) solution upon recovery and transferred to 80% ethanol at −20 °C for subsequent fluorescence in situ hybridisation (FISH) experiments.

### Endosymbiont nucleic acid preparation and genome sequencing

The endosymbiont-hosting oesophageal gland was dissected from the scaly-foot snails and used for DNA extraction using a DNeasy Blood and Tissue Kit (Qiagen, Hilden, Germany) following the manufacturer’s protocols. DNA quality was assessed through agarose gel electrophoresis and BioDrop μLITE (BioDrop, Cambridge, UK), and the optical density (OD) 260/280 and OD 260/230 were obtained. DNA quantity was assessed with a Qubit 3 Fluorometer (Thermo Fisher Scientific, MA, USA). For each individual, approximately 1 μg of DNA of the oesophageal gland (OD 260/280 of 1.8 and OD 260/230 of 2.0–2.2) was used for short-insert library (350 bp) and sequenced in a NovaSeq 6000 platform (Illumina, CA, USA) to generate sufficient paired-end reads (Table S1) with a length of 150 bp.

### Symbiont genome assembly, gene prediction and functional annotation

Trimmomatic version 0.39 [24] was used to trim the adaptors and low-quality bases from raw sequencing reads with the settings of “ILLUMINACLIP:adaptor.fa:2:30:10:2:keepBothReads LEADING:3 TRAILING:3 MINLEN:36”. Clean reads were used for symbiont genome assembly and binning. SPAdes version 3.14.1 [25] with settings of “--meta, -k 61, 81, 101, 121” and Megahit version 1.2.9 [26] with settings of “--k-list 75, 95, 115, 135” were used to assemble the genomes. MaxBin2 version 2.2.7 [27] with default settings and manual binning methods [28, 29] were used to isolate the endosymbiont genomes according to the sequencing coverage and the GC content of assembled contigs. The completeness and contamination of each symbiont genome were assessed using CheckM version 1.0.13 [30]. The contiguity (contig N50 value) of the assembled genome was determined using assemblathon_stats.pl (https://github.com/ucdavis-633bioinformatics/assemblathon2-analysis/blob/master/assemblathon_stats.pl). The best assemblies were used for subsequent analyses. The gene sequences of each genome assembly were predicted using Prokka version 1.14.6 [31] with default settings. The predicted protein sequences were searched against the Non-Redundant database in NCBI by using BLASTp version 2.10.0+ with an *E*-value cut-off of 1e-5. The results were further used for Gene Ontology annotation by using OmicsBox version 1.4.11 (BioBam, Valencia, Spain). The predicted protein sequences were annotated using the Kyoto Encyclopedia of Genes and Genomes (KEGG) Automatic Annotation Server [32] with bi-directional best hit methods of BLASTp to search the KEGG database. The functional Clusters of Orthologous Groups (COG) of the predicted protein sequences were identified using eggNOG-mapper version 5 [33].

### Phylogenetic analysis and positive selection analyses

Roary version 3.13.0 [34] was used to identify the pan genome amongst the predicted genes of the symbiont genome assemblies from different host individuals. The pan genome results were further used to identify the core genes existing in all 23 endosymbiont assemblies, the accessory genes present in at least two assemblies but not all assemblies, and the assembly-specific genes of endosymbionts from different vent fields. The single-copy core genes were used for phylogenetic reconstruction. The closest known relative of the endosymbiont, a Chromatiaceae bacterium, was used as the outgroup [35]. Protein sequences of each single-copy orthologous gene were aligned using MUSCLE version 3.8.31 [36]. The alignment of all single-copy core genes was further concatenated and used for phylogenetic reconstruction using IQ-TREE multicore version 1.6.12 [37] with the model LG + I + G4 + F and 1000 ultrafast bootstraps. The host mitochondrial phylogenetic tree was constructed based on 11 protein-coding genes following the same method of symbiont phylogenetic analysis. The Robinson–Foulds distance [38] between the symbiont and mitochondrial phylogenies was quantified using T-REX [39].

The coding DNA sequences (CDS) of 1681 single-copy core genes were aligned under the guidance of their alignments of protein sequences by using PAL2NAL version 14 with default settings to identify genes under positive selection [40]. Gblocks version 0.91b with settings of ‘-t=c’ [41] was used to trim the poorly aligned codons. On the basis of the phylogenetic tree, the CDS alignments of these single-copy genes were used to detect positive selection pressure using the MEME [42] and FUBAR [43] models implemented in HyPhy version 2.5.32 [44]. KEGG pathway enrichment analysis was performed based on the results of rich factor calculation and hypergeometric test. The rich factor of each pathway was calculated as the ratio of positively selected gene numbers annotated in a given KEGG pathway term to all gene numbers annotated in the given KEGG pathway, which indicates the degree of pathway enrichment. A hypergeometric test was used to determine the statistical significance, and FDR values < 0.05 were considered to indicate that the pathways were enriched with positive selection.

### Single-nucleotide polymorphisms (SNPs) and population variation analyses

To understand the population structures and transmission mode of the endosymbionts, we treated each host individual as an endosymbiont population to perform across-vent and within-vent population variation analyses. In the across-vent analysis, population variation analyses were identified and compared across individuals from all vent fields by using the symbiont genome published by Nakagawa et al. [15] as a reference genome for calling the genome-wide SNPs of endosymbiont populations in each snail. The whole genome sequencing reads were aligned to the reference genome by using Bowtie2 version 2.3.5 [45] coupled with SAMtools version 1.9 [46]. A pipeline of Genome Analysis Toolkit (GATK) version 4.1.9.0 [47] was used to recover the SNPs in the symbionts. In detail, the duplicated reads were filtered using GATK MarkDuplicates. SNPs were called using GATK HaplotypeCaller with settings of “ploidy = 6” (Table S2) to detect mixed bacterial populations [2]. GATK VariantFiltration was used to filter the unreliable SNPs with the settings of “QD < 2.0 ||  MQ < 40.0 ||  SOR > 4.0 ||  FS > 60.0 ||  MQRankSum < –12.5 ||  ReadPosRankSum < −8.0”. SnpEff version 5.0c [48] was used to annotate the SNPs and assess the effect of genetic variants. The SNPs of core genes were extracted using BCFtools version 1.12 [49]. The SNPs were used to examine the genetic divergence by using the R package SNPRelate version 1.28.0 [50]. The per-gene nucleotide diversity (*π*) of intra-host and inter-host endosymbiont populations and fixation index (*F*_*ST*_) values indicating the differentiation of genetic structure amongst different symbiont populations was analysed. The *π* and *F*_*ST*_ were calculated following the methods and codes (https://github.com/deropi/BathyBrooksiSymbionts/tree/master/Population_structure_analyses) implemented by Ansorge et al. [2]. Based on the *F*_*ST*_ values, the ordination for the PCoA plot was performed with the ‘pcoa’ function from the R package ape version 5.5 [51] to measure the divergence of symbiont populations amongst host individuals within vents, within ridges, and across ridges. Hierarchical distance-based redundancy analysis (db-RDA) [52] was performed with the “capscale” function from the R package vegan version 2.5-7 [53]. This analysis was further supported by PERMANOVA statistics on pairwise Bray–Curtis dissimilarities with 1000 permutations.

Considering that the symbiont populations showed significantly different genetic divergence patterns across the five vents (Fig. 1b and Fig. S1), we further measured the population genetic diversity within each vent using the endosymbiont genome with the largest contig N50 value of individuals in each vent as the reference genome for calling the SNPs. The SNPs of core genes were identified following the same pipeline used for analysis across vents. Principal component analysis (PCA) of intra-host and inter-host nucleotide diversity was performed for each vent using PAST version 4.03 [54], which was supported by PERMANOVA statistics on pairwise Bray–Curtis dissimilarities with 9999 permutations.

### Histology and FISH

Three ovarian and three testicular tissues from three individuals of scaly-foot snails (they are hermaphrodites) were dissected and embedded in Epredia histoplast paraffin (Thermo Fisher Scientific, MA, USA) using a Revos tissue processor (Thermo Fisher Scientific, MA, USA) with the standard program. Then, the tissues were mounted and embedded with a HistoStar embedding station (Thermo Fisher Scientific, MA, USA). Ten tissue sections (5 µm thickness) of each tissue were cut using HM325 rotary microtome (Thermo Fisher Scientific, MA, USA). For hematoxylin and eosin (HE) staining, the tissue sections were dewaxed and then stained with HE in accordance with the standard protocol. After staining, the sections were dehydrated and sealed with neutral balsam. The images were captured using an AxioScan slide scanner (Zeiss, Oberkochen, Germany). For FISH experiments, the tissue sections were first dewaxed in accordance with the standard protocol and then washed three times with PBST (1 × PBS, 0.1% Tween 20) for 5 min each. Specific probes based on a unique region of the 16S rRNA gene of the symbiont (5ʹ-GCGCCACTAAACCCGTAAATG-3ʹ) were designed for binding the symbiont specifically. The specificity of the probe was confirmed by searching against the RDP database with Probe Match [55]. Hybridisation with symbiont-Cy3 and EUB338-Cy5 probes [56] was conducted following the protocol described by Halary et al. [57]. In detail, tissue sections were hybridised at 46 °C for 3 h with hybridisation buffer (50 ng/mL of each probe, 0.9 M NaCl, 0.02 M Tris-HCl, 0.01% sodium dodecyl sulphate, and 20% formamide) and then washed in the buffer with 0.1 M NaCl, 0.02 M Tris-HCl, 0.01% sodium dodecyl sulphate, and 5 mM EDTA at 48 °C for 15 min. After the hybridisation washing, the sections were labelled with 4′,6-diamidino-2-phenylindole (DAPI, Sigma-Aldrich, MO, USA) and Alexa Fluor 488 Conjugate Concanavalin-A (Invitrogen, CA, USA). The sections were finally mounted on a Prolong glass antifade mounting medium (Invitrogen, CA, USA) and then imaged under a Nikon AX confocal microscope.

### Measurement of environmental factors in scaly-foot snail colonies

At Solitaire, colony water from the scaly-foot snail aggregations was collected using a Hachi-Ren water sampler [58] mounted on DSV *SHINKAI 6500*, and then a pistol-like sampler was inserted into the aggregation to collect water between the snails in the assemblage. The collected fluids were processed as quickly as possible after the recovery of DSV *SHINKAI 6500* to minimise the influences of light, oxygen, and temperature. Dissolved gas was extracted from the colony water samples as previously described [58]. The concentrations of dissolved H_2_ and CH_4_ were determined using gas chromatography with a helium ionisation detector (GC-HID, GL Science, CA, USA). The concentrations of sulphide and iron (Fe^2+^) were spectrophotometrically determined using the methylene blue method [59, 60] and the ferrozine method [61, 62], respectively. Water samples obtained using Niskin bottles approximately 400 m away from the venting activity were used as reference for ambient seawater. A phosphorescence dissolved oxygen (DO) and temperature sensor (ARO-USB, JFE Advantech Co. Ltd., Kobe, Japan) was used to measure the scaly-foot snail colony by directly placing the instrument over the snails in situ for 5 min.

Similarly measured environmental parameters for Kairei (with additional measurements using in situ sensors on DSV *SHINKAI 6500*) were published in Miyazaki et al. [23] and used herein, although the data for methane (R/V *Yokosuka* cruise YK09-13 Leg 2, November 2009) and Fe^2+^ (R/V *Yokosuka* cruise YK13-13), measured following the same methods as above, are newly reported here. Endmember fluid characteristics of the Longqi, Kairei, and Solitaire hydrothermal vents were estimated as previously described [58, 63–65].

### Gene expression quantification

The oesophageal glands of in situ RNAlater fixed scaly-foot snails were dissected and used for RNA extraction. The TRIzol reagent (Invitrogen, CA, USA) was used to extract total RNA, the quality of which was assessed through agarose gel electrophoresis. The Ribo-Zero Magnetic Kit (Human/Mouse/Rat) (Epicenter, WI, USA) and the Ribo-Zero Magnetic Kit (Bacteria) (Epicenter, WI, USA) were further used to remove the rRNA of both eukaryote and bacteria to enrich the mRNA of the symbionts. The enriched symbiont mRNA was used to construct the cDNA library for metatranscriptome sequencing using a NovaSeq 6000 platform (Illumina, CA, USA). Details of the resulting sequencing data are provided in Table S3. Salmon version 1.2.1 [66] was used to quantify the gene expression level of each gene. The differentially expressed genes of symbionts of host snails from Kairei and Solitaire were identified using Trimmed Mean of *M*-values normalisation method and edgeR package implemented in RNA-seq 2 G [67]. Differentially expressed genes were those with a fold change >2 or <0.5 and an FDR < 0.05. For the host gene expression analyses, different tissues of the same host individuals (Kairei: B2, B8, E02B1, and E02B2; Solitaire: IW1, IW2, IW3, W2, and W7) were used. Functional enrichment analysis was conducted following the same pipeline used in the positive selection analysis. PCA supported by PERMANOVA on pairwise Bray–Curtis dissimilarities with 9999 permutations was applied to analyse the normalised gene expression data using PAST version 4.03 [54]. The transcriptome sequencing reads of different tissues, including foot, ctenidium, sclerites, and oesophageal gland, were recruited from our previous studies [68] following the same pipelines used in the gene expression analyses of symbionts.

### TMT-labelling metaproteomic analysis

The oesophageal glands of scaly-foot snails from Kairei and Solitaire were used for protein extraction through the methanol–chloroform method [69]. The proteins extracted were resolved in a lysis buffer (8 M urea, 20 mM Tris-32 HCl, pH 8.0), and the quantity was measured using a Qubit 3 Fluorometer (Thermo Fisher Scientific, MA, USA), followed by the reduction and alkylation of the extracted proteins. Approximately 30 μg of protein from the oesophageal gland of each snail was digested by trypsin (Promega, WI, USA) and further desalted into peptide following the C18 cartridge solid-phase extraction (Sep-Pak, MA, USA). The peptides were subsequently labelled with TMT 10-plex reagents (TMT 10plex Isobaric Label Reagent Set, Thermo Fisher Scientific, MA, USA) following the manufacturer’s instructions. High-performance liquid chromatography (HPLC) fractionation of the TMT-labelled mix peptide was performed on a Rigol L3000 HPLC. An EASY-nLC 12-UHPLC system (Thermo Fisher Scientific, MA, USA) coupled with an Orbitrap Q Exactive HF-X mass spectrometer (Thermo Fisher Scientific, MA, USA) was used to perform shotgun proteomics. Proteome Discoverer v2.2 (Thermo Fisher Scientific, MA, USA) was used to identify and quantify the proteins through searching the TMT spectra against the target-decoy database of the scaly-foot snail holobiont with the settings of 10 ppm mass tolerance for the precursor ion and 0.02 Da mass tolerance for the product ion. Median normalisation and nonparametric rank product test were used to identify the differentially expressed proteins with a fold change > 1.5 or < 0.67 and an FDR < 0.05. The normalised protein abundances of the host and symbionts were used for PCA supported by PERMANOVA using PAST version 4.03 [54].

## Results and discussion

### Transmission mode

We assembled one symbiont genome and one host mitochondrial genome from each of the 23 scaly-foot snail individuals, including five from Kairei (including data of one individual from Nakagawa et al. [15] to make five), five from Solitaire, five from Longqi, five from Tiancheng, and three from Wocan (as only a few animals have been collected from this site so far) (Fig. 1 and Table S1). The size of the assembled endosymbiont genomes varied from 2.52 to 2.83 Mb, with 98.4–99.9% completeness (Table S1). Their average nucleotide identity (ANI) varied from 96.7 to 100%, and those from the same vent field had ANIs greater than 99.3% (Fig. S2). The endosymbionts from each vent field showed higher similarity amongst themselves compared with those from the other vent fields (e.g. between Tiancheng and the four other vent fields). The high ANI values indicated that the snail endosymbionts from the five vent fields belonged to a single symbiont species, considering that a minimum 95% ANI value is the threshold for bacterial species [70]. These results also suggested that the host species established its symbiosis with a specific species of symbiont, supported by the fact that different species of vent peltospirid snails have species-specific partners despite their side-by-side occurrence [17]. Phylogenetic analysis of the endosymbionts based on 1499 single-copy core genes (Fig. 1b) revealed a similar pattern, with the snail endosymbionts from the same vent field clustering together. However, the mitochondrial phylogenetic tree of the host snails showed that the host individuals from the four vent fields, except for Longqi, were mixed with no particular pattern, whereas the Longqi individuals formed a distinct clade (Fig. 1c). This result agrees with the findings of previous host population genetics studies [19, 20]. The endosymbiont lineages were clearly geographically segregated according to vent fields (Fig. 1b) but not to the host (Fig. 1c). The incongruent topologies and a topological Robinson–Foulds distance of 33 (i.e. 33 internal branches were present only in one tree and not in the other) between the host and symbiont phylogenies suggest that scaly-foot snails acquire endosymbionts through horizontal transmission from the environment at each generation [15]. Nevertheless, occasional horizontal transmission could break the congruence of the host mitochondrial and symbiont phylogenies of *T. magnifica* adopting nearly a strict vertical transmission mode [9]. Therefore, the possibility of vertical transmission cannot be excluded entirely.

**Fig. 2:**
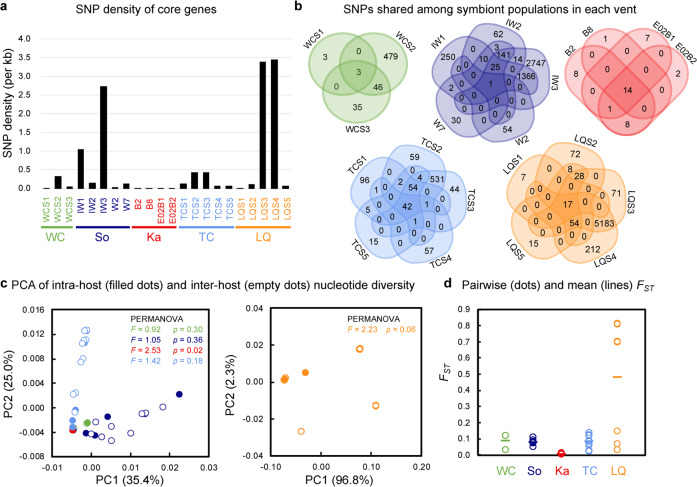
Symbiont population genetic measures of scaly-foot snails from five hydrothermal vents in the Indian Ocean, assessing within each vent. **a** SNP density of core genes of each symbiont population. **b** Venn diagrams of SNP shared among symbiont populations of all host individuals in each vent. **c** PCA of intra-host (filled dots) nucleotide diversity (*π* values) and pairwise inter-host (empty dots) nucleotide diversity based on core genes of symbiont genomes. Individuals from Longqi are separated in another PCA plot because their nucleotide diversities are much higher than those in the other vents. PCA was supported by PERMANOVA on pairwise Bray–Curtis dissimilarities with 9999 permutations. PERMANOVA between intra-host and pairwise inter-host nucleotide diversity within each vent: Wocan: pseudo-*F* = 0.92 and *p* value = 0.30; Solitaire: pseudo-*F* = 1.05 and *p* value = 0.36; Kairei: pseudo-*F* = 2.53 and *p* value = 0.02; Tiancheng: pseudo-*F* = 1.42 and *p* value = 0.18; Longqi: pseudo-*F* = 2.23 and *p* value = 0.06. **d** Pairwise (empty dots) and mean (lines) *F*_*ST*_ values of symbiont populations across all host individuals within each vent. *F*_*ST*_ values indicate differentiation due to genetic structure, when treating each host individual as a population of endosymbionts. Colour: Wocan (WC: green), Solitaire (So: dark blue), Kairei (Ka: red), Tiancheng (TC: light blue), and Longqi (LQ: orange). Source data are provided in a Source Data file.

For the population variant analysis across vents, db-RDA and PCA analyses based on the SNPs (Fig. S1) revealed that the genetic structures of symbiont populations were segregated by vent fields. This result is in line with the fact that the pairwise *F*_*ST*_ values of the two host individuals from different vents were higher than those of two individuals within the same vent, indicating that the across-vent genetic divergences were much greater than the within-vent divergence (Fig. S2). For the within-vent analysis, the SNP density and genetic diversities of core genes of each endosymbiont population showed that the extreme intra-host and inter-host genetic homogeneity (SNP density: 0.01–0.02 SNPs/kbp; *F*_*ST*_ value of pairwise inter-host symbiont populations: < 0.01) of endosymbionts was true for Kairei [15] but not for the other vent fields (Fig. 2, Table S4). In the four other vents, only a small part of the SNPs (Wocan: 3 shared SNPs/618 SNPs in total; Solitaire: 1 shared SNP/6,471 SNPs in total; Tiancheng: 42 shared SNPs/1,797 SNPs in total; Longqi: 17 shared SNPs/11,144 SNPs in total) was shared among all host individuals, suggesting that a considerable heterogeneity was exhibited across host individuals living in the same vent field (Fig. 2b). Except for IW1 and IW3 from Solitaire and LQS3 and LQS4 from Longqi, most symbiont populations within each host exhibited high homogeneity (SNP density: 0.004–0.44 SNPs/kbp) (Fig. 2a), suggesting that the symbionts were obtained via a restricted process. For symbiont populations from Longqi, the *F*_*ST*_ values between LQS3/LQS4 and LQS1/LQS2/LQS5 were very high (*F*_*ST*_: 0.69–0.81), indicating high heterogeneity of symbiont populations between hosts (Fig. 2d, Fig. S2). *Bathymodiolus* mussels from Mid-Atlantic Ridges can continuously acquire diverse symbiont strains throughout the host’s lifetime and show high intra-host genetic diversity and inter-host homogeneity in symbiont populations living in the same vent [2]. However, a different case was observed in scaly-foot snails. That is, most symbiont populations exhibited intra-host homogeneity and symbiont populations from Longqi vent exhibited inter-host heterogeneity. Although the evidence of free-living symbiont populations of scaly-foot snails in the environment is currently unavailable, the symbiont possesses highly similar genomic features, central metabolic pathways, and motility (i.e. flagellar genes and chemotaxis genes) to its closest known free-living relative, the Chromatiaceae bacterium CTD079 [35]. The symbionts of scaly-foot snails from the five vents possessed assembly sizes ranging from 2.52 to 2.83 Mb (Table S1), including a genome size of 2.60 Mb as reported by Nakagawa et al. 2014, close to that of the Chromatiaceae bacterium CTD079 (genome size: 2.75 Mb). These similar features between the symbiont and its free-living relatives suggest that the symbiont likely possesses a free-living stage. Therefore, scaly-foot snails likely acquire symbiont strains from the ambient environment in a restricted period of the life cycle or vertically inherited symbionts from parental gametes.

**Fig. 3:**
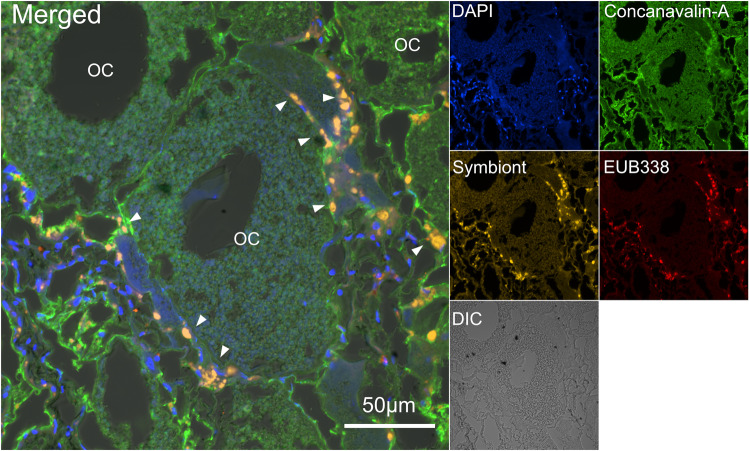
Fluorescence in situ hybridisation (FISH) image with specific probe based on the 16S rRNA gene yielding symbiont signals surrounding oocyte cells (OC) in the ovary tissue of a scaly-foot snail. White arrow: symbiont (in yellow), nuclear DNA with DAPI staining is blue, universal bacterial probe (EUB338) is in red.

The incongruent phylogeny and results of the genetic analyses suggest that scaly-foot snails likely adopted horizontal transmission to obtain its symbionts, but neither can eliminate the possibility of vertical transmission. Therefore, we conducted FISH imaging of the gonads to clarify the possibility of vertical transmission. Results revealed positive signals of the specific probe of the symbionts surrounding the oocytes (Fig. 3, Fig. S3a) but none in the testis (Fig. S3c). These results suggest that scaly-foot snails, similar to vesicomyid clams, are likely capable of transferring the symbionts to their offspring via eggs [6]. In the vesicomyid clam *P. okutanii* with vertical transmission, the symbionts are found on the surface of egg cells rather than intracellularly like many other vertically transmitted systems, such as *Wolbachia* in insects [71]. Given the possible vertical transmission, another potential scenario that can lead to the incongruent phylogeny is that several strains could be transmitted on the eggs and then only one strain is selected by the environment during post-settlement growth to dominate in the adult individual. However, as the scaly-foot snail endosymbiont likely possesses a free-living stage, this scenario is less likely than horizontal acquisition. Nevertheless, further studies to confirm the presence of the symbiont in the environment and sequencing of recently settled juvenile snails (not available in the present study) are warranted to rule out this possibility completely.

Collectively, the scaly-foot snails likely adopted a mixed transmission mode to establish their symbiosis. A mixed transmission mode occurs in many organisms, such as *Solemya* clams [8], enabling the host to take advantage of both modes [72]. Horizontal transmission could provide the host animals an opportunity to select symbionts that are well adapted and optimised for their specific local environments [13], whereas vertical transmission could provide advantages in mediating maintenance and building a strong fidelity between the symbiotic partners at the species level [73]. Therefore, we hypothesised that a combination of both modes could contribute to the success of scaly-foot snails in many Indian Ocean vents across a wide geographic range.

### Pan-genomes of symbiont populations

**Fig. 4:**
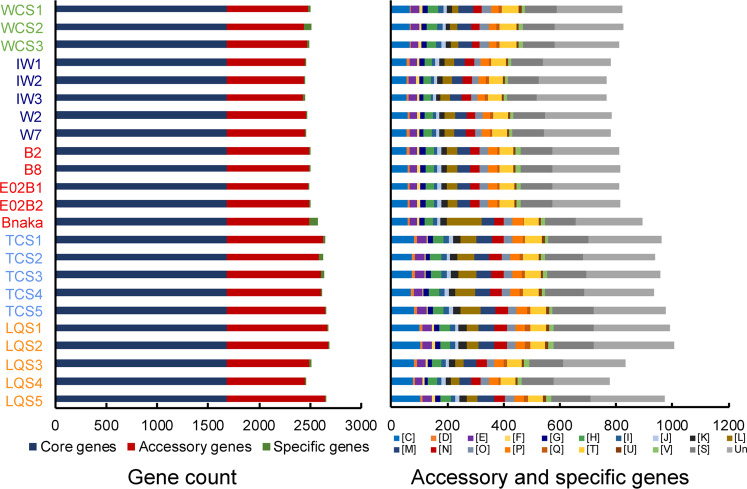
Overview showing the pan-genomes of endosymbionts of the 23 scaly-foot snails from the five vent fields. The left column figure shows the numbers of core genes, accessory genes, and assembly-specific genes of different assemblies. The right column figure shows the functional Clusters of Orthologous Groups categories of accessory and assembly-specific genes of 23 symbiont assemblies. Colour labelled in the host individuals: Wocan (green): WCS1, WCS2, and WCS3; Solitaire (dark blue): IW1, IW2, IW3, W2, and W7; Kairei (red): B2, B8, E02B1, E02B2, and Bnaka (Nakagawa et al. 2014 [15]); Tiancheng (light blue): TCS1, TCS2, TCS3, TCS4, and TCS5; Longqi (orange): LQS1, LQS2, LQS3, LQS4, and LQS5. [C] Energy production and conversion; [D] Cell cycle control, cell division, chromosome partitioning; [E] Amino acid transport and metabolism; [F] Nucleotide transport and metabolism; [G] Carbohydrate transport and metabolism; [H] Coenzyme transport and metabolism; [I] Lipid transport and metabolism; [J] Translation, ribosomal structure, and biogenesis; [K] Transcription; [L] Replication, recombination, and repair; [M] Cell wall/membrane/envelope biogenesis; [N] Cell motility; [O] Post-translational modification, protein turnover, and chaperones; [P] Inorganic ion transport and metabolism; [Q] Secondary metabolites biosynthesis, transport, and catabolism; [T] Signal transduction mechanisms; [U] Intracellular trafficking, secretion, and vesicular transport; [V] Defence mechanisms; [S] Function unknown; Un: Un-annotated. Source data are provided in a Source Data file.

A comparison of the composition of functional genes amongst all 23 symbiont assemblies revealed variations in the gene content and the statistics of core, accessory, and assembly-specific genes (Fig. 4, Supplementary Data 1). Amongst the 23 symbiont assemblies from the five vent fields, 62.6%–68.8% of the genes were core genes present in all symbiont assemblies; 30.0–36.9% were accessory genes that were present in at least two but not all assemblies; and only 0.002–0.03% were assembly-specific genes (Fig. 4). The core genes included those involved in the Calvin cycle for carbon fixation, TCA cycle, glycolysis, sulphur oxidation, and biosynthesis of essential amino acids and vitamins, which are the key functions for supporting the host snail. When performing positive selection detection across vents, the biosynthesis pathways of amino acids were enriched, indicating the selective force on nutritional commands of the holobionts under the different environmental conditions in the five vents (Fig. S4, Supplementary Data 2, Supplementary Note 1). Very few genes were found to be under positive selection within each vent, suggesting a relaxation of selection in the host individuals living in the same vent field.

**Fig. 5:**
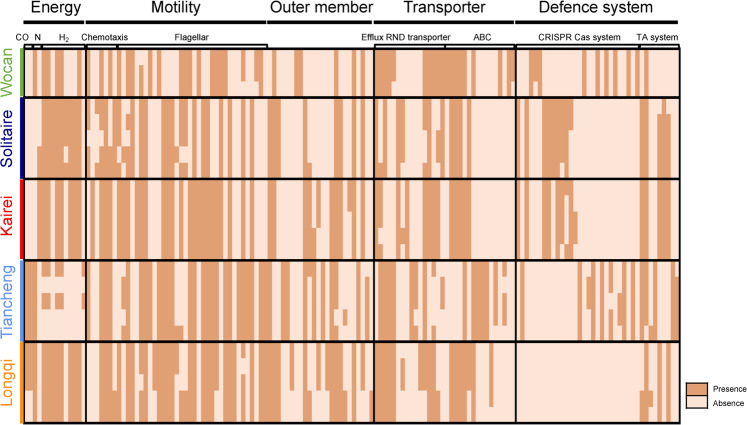
Metabolic variation in endosymbiont populations of scaly-foot snails from five different vent fields. Genes involved in energy metabolism, cell motility, transporters, and defence system exhibit content variations amongst symbiont populations. (CO: carbon monoxide metabolism, N: nitrogen metabolism, ABC: ABC transporters, TA: toxin-antitoxin). Source data are provided in a Source Data file.

Examination of the accessory genes amongst the 23 symbiont assemblies allowed us to further explore the metabolic variation based on functional COG categories amongst the symbionts. The accessory genes were mainly involved in several COG categories (shown as capital letters in square brackets), including energy production and conversion [C]; replication, recombination, and repair [L]; cell wall/membrane/envelope biogenesis [M]; cell motility [N]; signal transduction mechanisms [T]; and intracellular trafficking, secretion, and vesicular transport [U] (Fig. 4). Within the same vent field, similar patterns of gene contents were detected amongst endosymbionts of snails with a variation of only a few genes (Fig. 5). The patterns of gene contents were likely driven by local environmental selection because the accessory gene patterns were clearly segregated according to the vents (Fig. 5, Fig. S5, Supplementary Note 2). For example, the hydrogenase maturation protease was present in the Solitaire symbionts but absent in the Kairei symbionts, which is congruent with the fact that hydrogen concentration in Solitaire is much higher than that in Kairei (Table 1). Such variations in gene contents involved in essential energy metabolism may be associated with the availability of certain chemical resources in the local environments, indicating the selection forces on the gene content from the environmental conditions. The most energetically optimal stains likely dominated each vent field.

**Table 1:** Environmental factors of scaly-foot snail colonies in Kairei and Solitaire vents.

Parameter	Depth (m)	Temp (°C)	DO (μM)	H_2_S (μM)	H_2_ (μM)	Fe^2+^ (μM)	CH_4_ (μM)
Kairei	2422	8.4–13.5	133.0–187.0	23.5–29.6 [25.1]^#^ in situ	8.0–8.6 [8.4]^#^ in situ	16.6–20.0	6.5–11.5
		[12.6]^#^	[158.0]^#^	<1.5^#^ on-board	6.4–10.2 [8.2]^#^ on-board	[18.8]*	[8.7]*
Solitaire	2606	2.3–36.9	20.6–212.0	19.0–133.0	10.0–26.7	5.0–11.5	1.5–6.4
		[24.2]*	[47.4]*	[52.1]* on-board	[18.2]* on-board	[8.4]*	[4.2]*

* this study, ^#^ Miyazaki et al. 2020, [Average], *Temp* temperature, *DO* dissolved oxygen.

### In situ environmental factors and gene expression

Considering that scaly-foot snails inhabit vent fields with varying environmental conditions [58] and host different endosymbiont strains, we explored how the holobionts respond to different environmental conditions and whether the strains function differently across the sites. To address these questions, we compared the transcriptome data of scaly-foot snails from Kairei and Solitaire, the two fields where snails were fixed in situ, environmental factors of the snail colonies were measured either in situ using sensors or water samples collected in gas-tight tubes that were measured on-board, and symbiont populations were genetically close.

**Fig. 6:**
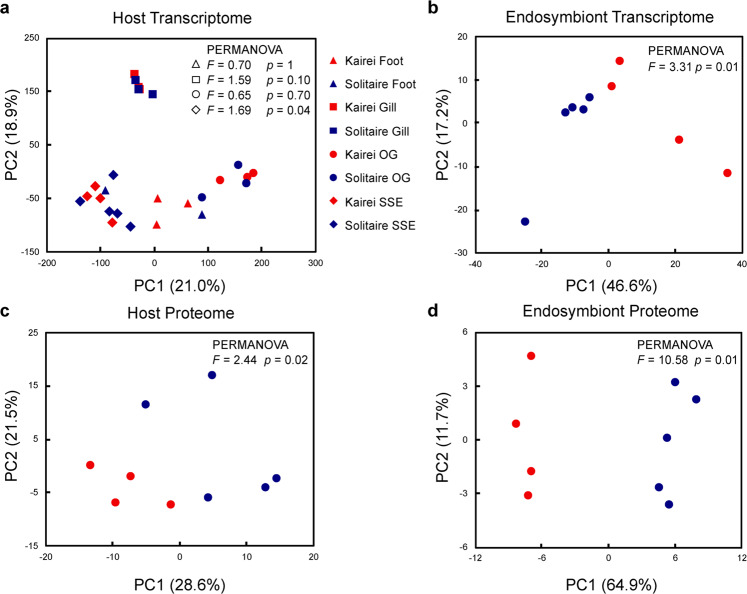
Comparative expression patterns of genes and proteins between the scaly-foot snail holobionts from Kairei and Solitaire vents. **a** Principal component analysis (PCA) of the normalised gene expression data for different tissue types of the host snail, including foot, gills, oesophageal gland (OG), and scale-secreting epithelium (SSE), from Kairei and Solitaire. PCA was supported by PERMANOVA on pairwise Bray–Curtis dissimilarities with 9999 permutations. Internal tissue of foot: pseudo-*F* = 0.70 and *p* value = 1; gill: pseudo-*F* = 1.59 and *p* value = 0.10; OG: pseudo-*F* = 0.65 and *p* value = 0.70; SSE: pseudo-*F* = 1.69 and *p* value = 0.04. **b** PCA of the normalised gene expression data of endosymbionts from Kairei and Solitaire. PERMANOVA pseudo-*F* = 3.31 and *p* value = 0.01. **c** PCA of the normalised protein abundance of host in the oesophageal gland from Kairei and Solitaire. PERMANOVA pseudo-*F* = 2.44 and *p* value = 0.02. **d** PCA of the normalised protein abundance of endosymbionts from Kairei and Solitaire fields. PERMANOVA pseudo-*F* = 10.58 and *p* value = 0.01. Source data of (**a**), (**b**), (**c**), and (**d**) are provided in a Source Data file.

**Fig. 7:**
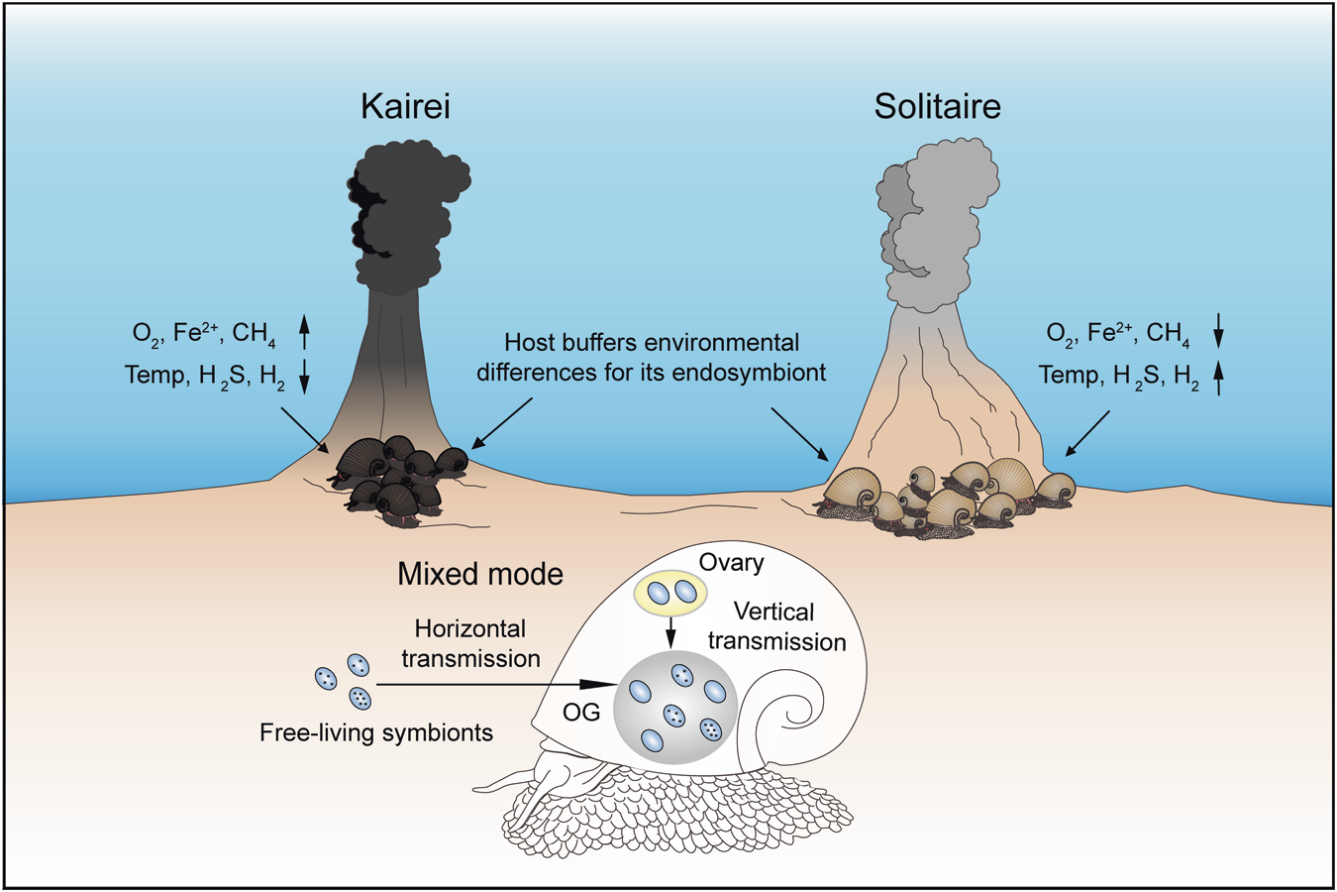
Schematics of the transmission mode, genetic diversity of symbionts, and the environmental differences buffered by the host. Symbionts without or with different numbers of dots represent symbionts with different genetic variants. OG: oesophageal gland.

Local environmental factors of the scaly-foot snail colonies, including temperature and the concentrations of DO, hydrogen sulphide, hydrogen, methane, and iron (Fe^2+^), varied in Kairei and Solitaire (Table 1). Such variation is expected for vent ecosystems [74]. Temperature, DO content, and hydrogen sulphide content exhibited higher variations in Solitaire than in Kairei. In general, higher concentrations of hydrogen and hydrogen sulphide but lower concentrations of methane and iron were found in Solitaire than in Kairei (Table 1). Although hydrogen sulphide was only measured on-board for Solitaire and autooxidation during recovery cannot be excluded, the on-board values for Solitaire were still higher than the in situ measurements in Kairei, indicating that hydrogen sulphide concentration is indeed higher in Solitaire than in Kairei. The compositions of endmember fluids in these two vents exhibit considerable differences [58]. These results showed that the scaly-foot snails from the two vent fields had different local environments and fluctuation levels, as evidenced by their different levels of iron sulphide mineralisation and colouration (Fig. 1b) [75]. The comparative gene expression of host genes significantly differed in the scale-secreting epithelia and the gills of the scaly-foot snails from the two vent fields, but no significant differences were observed in the endosymbiont-hosting oesophageal gland. This result indicates that environmental conditions exerted a significantly larger influence on the host gene expression of the tissues in direct contact with the environments than on that of the internal symbiont-hosting organ (Fig. 6a, Supplementary Data 3). For the endosymbionts, 122 genes were differentially expressed, but the KEGG enrichment analysis showed that no KEGG pathways were significantly enriched with differentially expressed genes (Fig. 6b, Supplementary Data 3). A few genes related to the biosynthesis of secondary metabolites, two-component system, and ABC transporters were differentially expressed, which were further supported by the protein abundance of the host and symbionts in the oesophageal gland (Fig. 6c, d, Supplementary Data 4). Notably, two genes related to sulphur metabolism, *soxZ* and *sqr*, were differentially expressed in the symbionts from the two vent fields. The symbionts showed a more similar symbiotic function in the scaly-foot snails living in the two vent fields than in the symbionts of *Alviniconcha* snails, which exhibited dynamic metabolism and gene expression with 1776 differentially expressed genes, including 17 key genes related to sulphur metabolism under different environmental conditions [76]. However, *Alviniconcha* snails host symbionts in the gill, which is in direct contact with the ambient environment; therefore, the host cannot regulate a stable intracellular environment. By hosting the endosymbiont in an organ deep inside the body, scaly-foot snails can regulate and likely maintain a steady concentration of key chemicals, such as hydrogen sulphide and oxygen [16]. In addition, they require a high specificity of endosymbionts at the species level to constrain its function in this specific intracellular habitat, whereas *Alviniconcha* retains flexibility in species-level associations [77]. Our results are congruent with a previous physiology experiment where scaly-foot snails can maintain a steady metabolic demand in two different experimental temperatures where *Alviniconcha* failed to do so [78]. Hence, scaly-foot snails can buffer shifts in the environmental conditions for the endosymbionts, a trait likely supported by their hypertrophied circulation system [16]. Overall, these results suggest that the scaly-foot snail hosts actively buffer the environmental differences and changes by moderating their gene expression to provide a stable intracellular environment in the oesophageal gland to the symbionts. Our findings on this symbiosis are summarised in Fig. 7.

## Conclusions

Scaly-foot snails likely adopt a combination of horizontal and vertical transmission modes to establish their symbiosis. Under this mixed transmission mode, scaly-foot snails can not only associate with the free-living symbionts that are well-adapted and optimised for their specific local environment, but also maintain a strong fidelity of symbiotic partners at the species level through heredity and ensure that their offspring can always acquire a suitable endosymbiont. Scaly-foot snails have evolved as an ‘armoured carrying vessel’ for the symbiont, with numerous adaptive novelties that ultimately contribute to their success in hosting endosymbionts and providing them with a stable environment inside its own body [16, 75, 78]. The symbiont benefits from having a protected, stable intracellular environment to grow and multiply, where they steadily provide core functions retained in all local strains for the host snails. The environmental conditions in each vent field select for energetically suitable strains, which all retain the core functions for endosymbiosis. The capacity of the snails to utilise vent-specific strains allows them to inhabit different vent fields. These features collectively underpin the success of the scaly-foot snail holobionts in different vent fields across the Indian Ocean, each with its own extreme environmental parameters.

## Supplementary information

Supplementary Material

Supplementary Data 1

Supplementary Data 2

Supplementary Data 3

Supplementary Data 4

Supplementary Data 5

## Data Availability

All genomic, transcriptomic and proteomic data were deposited in NCBI under BioProject PRJNA764822 and figshare 10.6084/m9.figshare.19882906.v1.

## Source data

Source Data

## Code availability

All computer commands are based on the available software and scripts listed in the Materials and Methods section and provided in Supplementary Data 5.
