# Supplementary material for: Endosymbiont population genomics sheds light on transmission mode, partner specificity, and stability of the scaly-foot snail holobiont
Source: ISME J. 2022 Jun 17;16(9):2132–43. doi: 10.1038/s41396-022-01261-4 (PMC9381778; doi:10.1038/s41396-022-01261-4)
Supplement: Supplementary file 1 — Supplementary Material [file 41396_2022_1261_MOESM1_ESM.docx]

**Table S1** Statistics of *Chrysomallon squamiferum* endosymbiont genome assembly and binning.

| **Vent Site** | **Host individual** | **Clean reads** | **Genome size (Mb)** | **Contig number** | **Completeness (%)** | **Contamination (%)** | **Genes** | **Read coverage (X)** |
| --- | --- | --- | --- | --- | --- | --- | --- | --- |
| **Kairei** | Bnaka | Nakagawa et al. 2014 | 2.60 | 1 | 98.90 | 0.64 | 2581 | **-** |
|  | B2 | 138,113,232 | 2.57 | 46 | 98.90 | 0.64 | 2496 | 4322 |
|  | B8 | 136,034,042 | 2.57 | 43 | 98.90 | 0.64 | 2498 | 5069 |
|  | E02B1 | 127,581,354 | 2.56 | 41 | 98.90 | 0.64 | 2495 | 4117 |
|  | E02B2 | 164,185,738 | 2.57 | 40 | 98.90 | 0.64 | 2500 | 5018 |
| **Solitaire** | IW1 | 159,273,954 | 2.53 | 56 | 98.55 | 0.81 | 2467 | 5418 |
|  | IW2 | 136,444,442 | 2.53 | 72 | 98.78 | 0.90 | 2454 | 4028 |
|  | IW3 | 144,830,372 | 2.52 | 79 | 98.43 | 1.16 | 2453 | 3915 |
|  | W2 | 133,194,208 | 2.55 | 60 | 99.25 | 0.81 | 2470 | 4648 |
|  | W7 | 130,110,444 | 2.54 | 54 | 99.13 | 1.16 | 2469 | 4841 |
| **Longqi** | LQS1 | 127,092,414 | 2.83 | 11 | 99.94 | 2.03 | 2676 | 2256 |
|  | LQS2 | 149,839,970 | 2.83 | 23 | 99.94 | 2.03 | 2690 | 3299 |
|  | LQS3 | 127,261,432 | 2.64 | 93 | 99.25 | 1.51 | 2516 | 2411 |
|  | LQS4 | 117,273,066 | 2.59 | 74 | 98.90 | 1.16 | 2461 | 1557 |
|  | LQS5 | 136,042,504 | 2.8 | 12 | 99.94 | 2.38 | 2655 | 1237 |
| **Tiancheng** | TC1S | 147,777,078 | 2.78 | 48 | 99.94 | 0.23 | 2646 | 3113 |
|  | TC2S | 171,123,514 | 2.75 | 94 | 99.94 | 0.93 | 2625 | 4145 |
|  | TC3S | 156,680,372 | 2.77 | 83 | 99.94 | 0.70 | 2644 | 4021 |
|  | TC4S | 164,514,052 | 2.74 | 45 | 99.94 | 0.93 | 2618 | 4186 |
|  | TC5S | 118,965,720 | 2.79 | 50 | 99.94 | 0.23 | 2661 | 3133 |
| **Wocan** | WC1S | 98,402,182 | 2.57 | 165 | 98.46 | 0.99 | 2507 | 2864 |
|  | WC2S | 120,189,096 | 2.56 | 227 | 98.46 | 0.99 | 2511 | 2868 |
|  | WC3S | 149,077,668 | 2.57 | 176 | 98.46 | 0.99 | 2496 | 3690 |

**Table S2** SNP densities of the intra-host symbiont population of three host individuals from each vent, with a series GATK ploidy settings test. In the present study, ploidy setting 6 was used for population variant analysis.

| **Vent** | **Individual** | **SNP density (p2)** | **SNP density (p4)** | **SNP density (p6)** | **SNP density (p10)** |
| --- | --- | --- | --- | --- | --- |
| **Wocan** | WCS1 | 0.002 | 0.003 | 0.004 | 0.005 |
|  | WCS2 | 0.339 | 0.339 | 0.340 | 0.340 |
|  | WCS3 | 0.049 | 0.054 | 0.054 | 0.054 |
| **Solitaire** | IW2 | 0.156 | 0.159 | 0.163 | 0.164 |
|  | W2 | 0.018 | 0.034 | 0.035 | 0.036 |
|  | W7 | 0.128 | 0.135 | 0.135 | 0.135 |
| **Kairei** | B8 | 0.010 | 0.010 | 0.010 | 0.010 |
|  | E02B1 | 0.014 | 0.014 | 0.014 | 0.014 |
|  | E02B2 | 0.016 | 0.016 | 0.016 | 0.016 |
| **Tiancheng** | TCS1 | 0.127 | 0.128 | 0.128 | 0.128 |
|  | TCS2 | 0.437 | 0.439 | 0.441 | 0.442 |
|  | TCS4 | 0.065 | 0.065 | 0.065 | 0.065 |
| **Longqi** | LQS2 | 0.113 | 0.113 | 0.113 | 0.113 |
|  | LQS3 | 3.370 | 3.375 | 3.375 | 3.375 |
|  | LQS4 | 3.445 | 3.446 | 3.446 | 3.446 |

**Table S3** Summary of metatranscriptome sequencing reads of scaly-foot snails from Kairei and Solitaire hydrothermal vents.

| **Site** | **Host individual** | **Clean Reads** |
| --- | --- | --- |
| **Kairei** | B2 | 69,556,176 |
|  | B8 | 73,441,912 |
|  | E02B1 | 100,016,364 |
|  | E02B2 | 69,910,610 |
| **Solitaire** | IW1 | 71,362,930 |
|  | IW2 | 74,365,288 |
|  | IW3 | 68,447,312 |
|  | W2 | 70,218,562 |
|  | W7 | 77,669,146 |

**Table S4** Summary of SNP density and nucleotide diversity of 1684 core genes of symbiont populations of scaly-foot snail. GATK ploidy setting is 6. Nucleotide diversity: *π*.

| **Site** | **Individual** | **SNP number** | **SNP density (per kb)** | **Missense/Silent ratio** | **Missense number** | **Missense density (per kb)** | **Intra-host***π* | **Intra-host** *π* **in each vent (average)** | **Pairwise inter-host** *π* **in each vent (average)** |
| --- | --- | --- | --- | --- | --- | --- | --- | --- | --- |
| **Kairei** | B2 | 31 | 0.020 | 2.875 | 23 | 0.015 | 5.24E-06 | 1.46E-06 | 6.84E-06 |
|  | B8 | 15 | 0.010 | 2.750 | 11 | 0.007 | 1.05E-08 |  |  |
|  | E02B1 | 22 | 0.014 | 2.143 | 15 | 0.010 | 2.16E-07 |  |  |
|  | E02B2 | 25 | 0.016 | 2.571 | 18 | 0.011 | 3.57E-07 |  |  |
| **Solitaire** | IW1 | 1,654 | 1.053 | 0.671 | 664 | 0.423 | 1.65E-04 | 1.58E-04 | 2.07E-04 |
|  | IW2 | 256 | 0.163 | 1.780 | 162 | 0.103 | 6.76E-05 |  |  |
|  | IW3 | 4,294 | 2.734 | 0.715 | 1,789 | 1.139 | 5.08E-04 |  |  |
|  | W2 | 55 | 0.035 | 1.667 | 35 | 0.022 | 1.10E-05 |  |  |
|  | W7 | 212 | 0.135 | 1.573 | 129 | 0.082 | 3.66E-05 |  |  |
| **Longqi** | LQS1 | 24 | 0.015 | 2.429 | 17 | 0.011 | 4.53E-06 | 1.61E-04 | 1.91E-03 |
|  | LQS2 | 179 | 0.113 | 1.712 | 113 | 0.071 | 2.66E-05 |  |  |
|  | LQS3 | 5,353 | 3.375 | 0.523 | 1,840 | 1.160 | 7.73E-04 |  |  |
|  | LQS4 | 5,466 | 3.446 | 0.512 | 1,851 | 1.167 | 1.11E-06 |  |  |
|  | LQS5 | 122 | 0.077 | 1.392 | 71 | 0.045 | 7.90E-08 |  |  |
| **Tiancheng** | TCS1 | 201 | 0.128 | 1.500 | 120 | 0.076 | 5.57E-05 | 8.97E-05 | 1.43E-04 |
|  | TCS2 | 694 | 0.441 | 1.753 | 440 | 0.279 | 1.95E-04 |  |  |
|  | TCS3 | 677 | 0.430 | 1.703 | 424 | 0.269 | 1.72E-04 |  |  |
|  | TCS4 | 102 | 0.065 | 1.615 | 63 | 0.040 | 6.09E-06 |  |  |
|  | TCS5 | 123 | 0.078 | 1.480 | 74 | 0.047 | 2.01E-05 |  |  |
| **Wocan** | WCS1 | 6 | 0.004 | 0.200 | 1 | 0.001 | 9.09E-07 | 5.98E-05 | 1.23E-04 |
|  | WCS2 | 528 | 0.340 | 1.875 | 345 | 0.222 | 1.71E-04 |  |  |
|  | WCS3 | 84 | 0.054 | 1.625 | 52 | 0.033 | 7.81E-06 |  |  |

**Figure S1 Population variation of the endosymbionts from five vents**. **a.** PCA revealing a genetic divergence pattern of five symbiont populations amongst five vent fields. The divergence pattern is further supported by a heat map of *F_ST_* values (see in **Figure S2**). **b.** Distance-based redundancy analyses (db-RDAs) test on the divergence of symbiont populations amongst host individuals within vents, within ridges and across ridges. PERMANOVA symbiont populations within vents: pseudo-*F* value < 1 and *p* value < 0.001; symbiont populations within ridges: pseudo-*F* = 21 and *p* value = 0.001; symbiont populations across ridges: pseudo-*F* = 9 and *p* value = 0.001. Colour labelling: Wocan (green), Solitaire (dark blue), Kairei (red), Tiancheng (light blue), and Longqi (orange). The source data of **b** are provided in a Source Data file.

**Figure S2 Heat map shows the average nucleotide identity (ANI, bottom left, in orange) compared amongst 23 assemblies and endosymbiont fixation index (*F_ST_*) values (top right, in blue), indicative of differentiation of genetic structure, when treating each host individual as a population of endosymbionts.**

**Figure S3** **a.** Fluorescence *in situ* hybridisation (FISH) image yielding signals of symbiont (yellow) surrounding the oocyte cells on transverse sections of the ovary tissue of a scaly-foot snail from Solitaire vent. **b.** Image of haematoxylin–eosin (HE) staining of the ovary tissue, showing oocyte cells recognized by large germinal vesicles (while spot). **c.** FISH images of the testis tissue of a scaly-foot snail from Solitaire vent. Nuclear DNA with DAPI staining is blue.

**
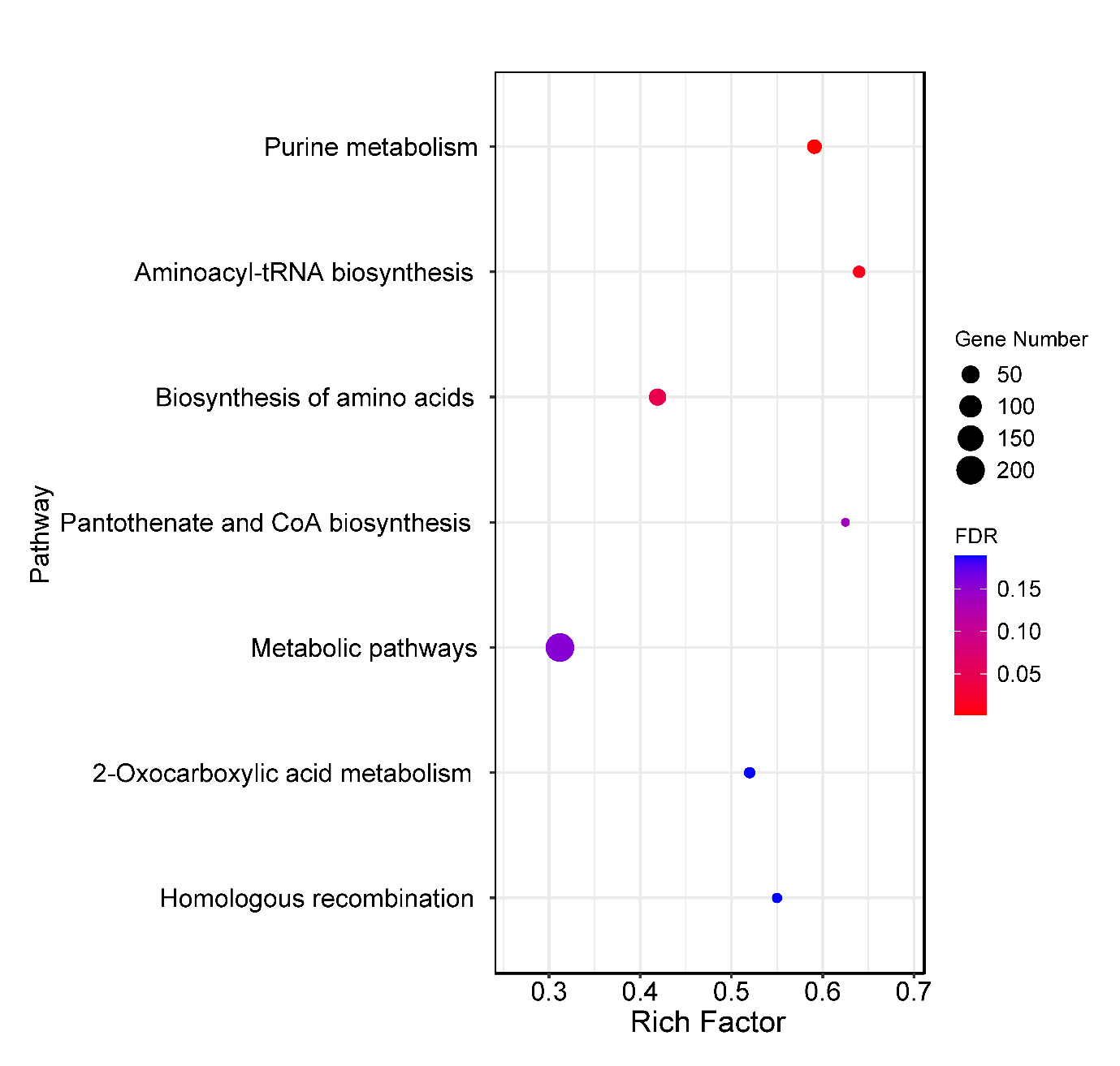
**

**Figure S4 A scatter plot for KEGG pathway enrichment of symbiont genes under diversifying positive selection.** The rich factor of each pathway is the ratio of positively selected gene numbers annotated in given KEGG pathway to all gene numbers annotated in the given KEGG pathway, which indicating the degree of pathway enrichment. The greater the rich factor, the greater the pathway enrichment. The source data is provided in a Source Data file.

**Figure S5 PCA based on the presence and absence pattern of accessory genes shows the symbiont populations are segregated by vents.** PERMANOVA statistics: TC-Ka: *p* value = 0.01 and pseudo-*F* = 952; TC-So: *p* value = 0.01 and pseudo-*F* = 583; TC-LQ: *p* value = 0.01 and pseudo-*F* = 62; TC-WC: *p* value = 0.02 and pseudo-*F* = 415; Ka-So: *p* value = 0.01 and pseudo-*F* = 87; Ka-LQ: *p* value = 0.01 and pseudo-*F* = 27; Ka-WC: *p* value = 0.02 and pseudo-*F* = 681; So-LQ: *p* value = 0.01 and pseudo-*F* = 28; So-WC: *p* value = 0.02 and pseudo-*F* = 270; LQ-WC: *p* value = 0.02 and pseudo-*F* = 22. Colour labelling: Wocan (WC: green), Solitaire (So: dark blue), Kairei (Ka: red), Tiancheng (TC: light blue), and Longqi (LQ: orange).

**Figure S6. Genes with functional divergence among the endosymbionts of the scaly-foot snail in the five vent fields based on a hidden Markov model (HMM)-based approach delta-bitscore (DBS)** [1]. **a.** Number of genes with functional divergence among symbiont populations in different COG categories. **b.** Flagellar genes showed significant divergence across the five hydrothermal vents. **c.** Chemotaxis genes showed significant divergence across the five hydrothermal vents. Colour labelling: Wocan (green), Solitaire (dark blue), Kairei (red), Tiancheng (light blue), and Longqi (orange).

**Figure S7** **The relations between symbiont genome sizes of 23 host individuals (horizontal axis) and their gene numbers (vertical axis), including the number of core genes, accessory genes, specific genes, and total genes.**

**Figure S8** **SNP density of core genes and accessory genes of each symbiont population, which are assessed within each vent.** Colour labelling: Wocan (green): WCS1, WCS2, and WCS3; Solitaire (dark blue): IW1, IW2, IW3, W2, and W7; Kairei (red): B2, B8, E02B1, and E02B2; Tiancheng (light blue): TCS1, TCS2, TCS3, TCS4, and TCS5; and Longqi (orange): LQS1, LQS2, LQS3, LQS4, and LQS5.

**
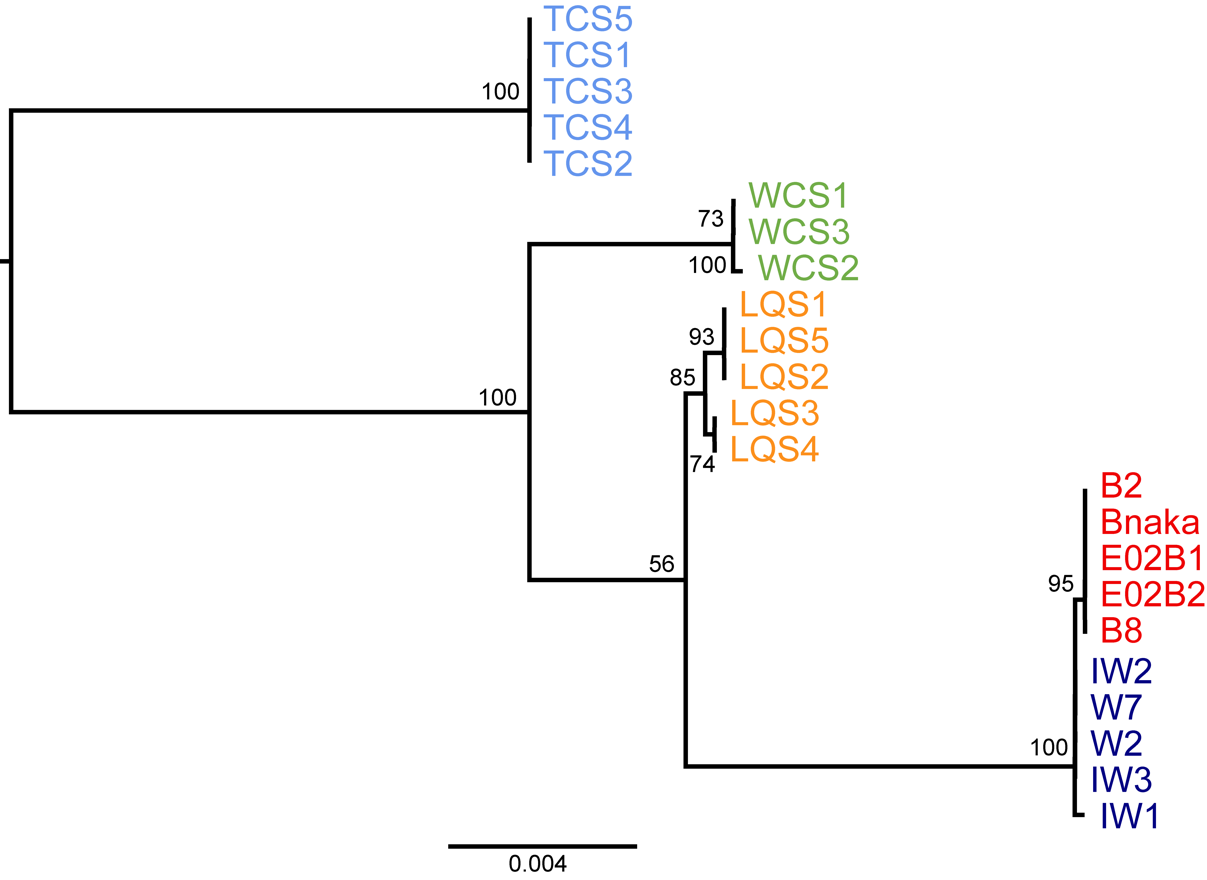
**

**Figure S9** **A phylogenetic tree based on 40 single-copy core genes without SNP of scaly-foot snail endosymbionts from the five vent fields.** Colour labelling: Wocan (green): WCS1, WCS2, and WCS3; Solitaire (dark blue): IW1, IW2, IW3, W2, and W7; Kairei (red): B2, B8, E02B1, E02B2, and Bnaka; Tiancheng (light blue): TCS1, TCS2, TCS3, TCS4, and TCS5; Longqi (orange): LQS1, LQS2, LQS3, LQS4, and LQS5.

**Supplementary Note 1**

**Positive Diversifying Selection of Genes Across Symbiont Populations**

For across-vents identification of positive selection, a total of 714 core genes were detected to be under diversifying positive selection pressure amongst the 23 endosymbiont assemblies from the five vent fields (**Supplementary Data 2**). Notably, the biosynthesis pathways of amino acids was enriched with genes under positive selection (**Fig. S4**). The selective force on nutrient biosynthesis may be derived from the nutritional demands of the symbionts in the free-living stage. If endosymbionts could be released as free-living form after host death, as is known from tubeworms [2], the selection force may also come from the nutritional demands of holobiont in the symbiotic stage. Both flagellar genes and chemotaxis genes showed significant geographical divergence (**Fig. S6**), indicating functional divergence of cell motility amongst local symbiont populations – crucial not only for avoiding lethal conditions in the fluctuating environments [3] but also for host invasion [4]. Given that microbial activity and community at vents are strongly constrained by the fluid chemistry and the underlying geological condition [5], these results indicated that the symbiont strains may be the energetically fittest ones selected by the local environmental conditions and adapted to each vent field, especially at the free-living stage.

**Supplementary Note 2**

**Variations in Gene Contents Amongst Symbiont Populations**

The symbionts from different vents differed in the presence of genes involved in the utilisation of carbon monoxide, hydrogen, nitrate, and nitrite (**Fig. 5**). For example, carbon monoxide dehydrogenase was present in the Tiancheng symbiont population and three individuals from Longqi, so these symbionts could utilise carbon monoxide as a carbon source. Nitrite reductase and nitrous-oxide reductase that are all involved in nitrogen metabolism also showed variation in gene content, indicating variation in the ability to use nitrate or nitrite as an electron acceptor. Furthermore, gene clusters for hydrogen oxidation, such as HypC/HybG/HupF family hydrogenase formation chaperone, HyaD/HybD family hydrogenase maturation endopeptidase, and hydrogenase maturation protease, showed variation amongst vent fields.

There were different gene counts of the ABC transporters for transporting amino acids amongst the endosymbionts, but a similar pattern of gene counts was present for those from the same vent field. This finding suggested different abilities of endosymbionts in exchanging nutrients and small molecules with environment in their free-living stage and possibly with the host in their endosymbiotic stage. The transporters related to metal, such as efflux RND transporter permease, also differed amongst symbiont assemblies, indicating a variation in their potential ability to detoxify metals for both the host and endosymbionts, perhaps adapting to the local conditions of each vent field. The symbionts also had various CRISPR-Cas systems and toxin–antitoxin systems (**Fig. 5**), indicating their different capabilities in interacting with local viruses and defence against viral infection.

**References**

1. Wheeler NE, Barquist L, Kingsley RA, Gardner PP. A profile-based method for identifying functional divergence of orthologous genes in bacterial genomes. Bioinformatics. 2016;32:3566-3574.

2. Klose J, Polz MF, Wagner M, Schimak MP, Gollner S, Bright M. Endosymbionts escape dead hydrothermal vent tubeworms to enrich the free-living population. Proc Natl Acad Sci USA. 2015;112:11300-11305.

3. Dick GJ. The microbiomes of deep-sea hydrothermal vents: distributed globally, shaped locally. Nat Rev Microbiol. 2019;17:271-283.

4. Nakagawa S, Shimamura S, Takaki Y, Suzuki Y, Murakami S, Watanabe T, et al. Allying with armored snails: the complete genome of gammaproteobacterial endosymbiont. ISME J. 2014;8:40-51.

5. Nakamura K, Takai K. Theoretical constraints of physical and chemical properties of hydrothermal fluids on variations in chemolithotrophic microbial communities in seafloor hydrothermal systems. Prog Earth Planet Sci. 2014;1:5.
